# Insights into Cdc13 dependent telomere length regulation

**DOI:** 10.18632/aging.100211

**Published:** 2010-10-23

**Authors:** Mark Mason, Emmanuel Skordalakes

**Affiliations:** The Wistar Institute, Gene Expression and Regulation program, 3601 Spruce St, Philadelphia, PA 19103, USA

**Keywords:** Cell division, telomeres, telomerase, aging, cancer

## Abstract

Cdc13 is a single stranded telomere binding protein that specifically localizes to the telomere ends of budding yeasts and is essential for cell viability. It caps the ends of chromosomes thus preventing chromosome end-to-end fusions and exonucleolytic degradation, events that could lead to genomic instability and senescence, the hallmark of aging. Cdc13 is also involved in telomere length regulation by recruiting or preventing access of telomerase to the telomeric overhang. Recruitment of telomerase to the telomeres for G-strand extension is required for continuous cell division, while preventing its access to the telomeres through capping the chromosome ends prevents mitotic events that could lead to cell immortality, the hall mark of carcinogenesis. Cdc13 and its putative homologues human CTC1 and POT1 are therefore key to many biological processes directly associated with life extension and cancer prevention and can be viewed as an ideal target for cancer and age related therapies.

## INTRODUCTION

Telomeres are short DNA repeats added to the ends of chromosomes by the reverse transcriptase telomerase [1,2], a ribonucleoprotein enzyme that uses an integral RNA template for DNA replication [3]. Telomeres prevent loss of genetic information, arising due to the “end replication problem” and protect the linear ends of chromosomes from recombination events and exonucleolytic degradation, all of which could lead to genomic instability and cell cycle arrest or death. Telomeres accomplish this task together with proteins that work exclusively at the ends of chromosomes to prevent such deleterious events and maintain cell viability. One of these proteins in budding yeast is Cdc13, which associates with single stranded telomeric DNA with high affinity and specificity [4,5]. Cdc13's ability to protect the ends of chromosomes is mediated in concert with the proteins Stn1 and Ten1 which together assemble into what is commonly known as the CST [Cdc13/Stn1/Ten1] complex [6,7] and which shares structural similarities with the replication protein A (RPA) complex [8]. Cdc13 further maintains the integrity of the chromosome ends by recruiting a number of factors, including telomerase and the DNA polymerase α (pol α), both of which are required for DNA replication [9-11]. It is well established that telomere elongation by telomerase takes place in late S to the G2 phase of the cell cycle [12,13] and when the G-rich overhang is of sufficient length for proper telomerase holoenzyme assembly [14,15].

Until recently, the Cdc13 dependent mechanism of chromosome end protection and telomerase regulation was thought to be unique to budding yeast and that higher eukaryotes including human were using a different set of proteins, known as the sheltering complex, for this purpose. For example, POT1, also a single stranded telomere binding protein, protects the chromosome ends (by suppressing the activity of the DNA damage response kinase ATR [16]), and negatively regulates telomerase activity [17] while its interacting partner, TPP1 recruits telomerase to the chromosome ends for telomere elongation [18,19]. However, it was not until recently that a series of studies identified the presence of the CST complex in several higher eukaryotes [8], suggesting that the CST mechanism of chromosome maintenance is unified across species and that the shelterin and CST complexes work in concert to maintain the integrity of the ends of chromosomes.

Because of its key role in cell viability, Cdc13 has been the focus of intense study. These efforts have produced a wealth of information regarding the complex biological function of the CST complex. However, a number of key questions underlying the mechanism of Cdc13 dependent telomere length regulation remain to be addressed. For example, it is not clear how Cdc13 regulates telomerase access to the telomeres. Cdc13 protects the very end of the chromosome which telomerase needs to bind to in order to begin telomere replication. What are the mechanistic events that surround this complex process? Significant insights into this mechanism were provided by the recent publication of structural, biochemical and functional data on the N-terminal domain of Cdc13 (Cdc13N:OB1), a domain thought to comprise the telomerase activation domain of the protein.

### Telomere Capping

**Figure 1.**
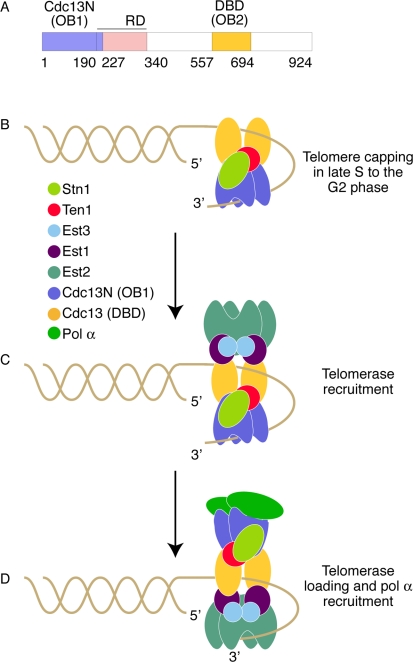
Model of Cdc13 dependent telomere length regulation. (**A**) Primary structure of Cdc13 showing the distinct domains in color. (**B**) CST dependent telomere capping during late S to G2 phase. The DBD and Cdc13N domains of Cdc13 are shown in yellow and blue color respectively. Stn1 and Ten1 are shown in lemon and red color respectively. (**C**) Telomerase (green claw) recruitment to the telomeres by Cdc13. The telomerase proteins Est1 and Est3 are shown in purple and cyan color respectively. (**D**) Telomerase loading and pol α (green oval) recruitment to the telomeres.

Cdc13 was identified in the early 1980s [20] and was later shown that it binds to long single stranded telomeric overhangs present during the late S to G2 phase, as well as to short telomeric overhangs present in the rest of the cell cycle [5]. The ability of Cdc13 to associate with the telomeric overhang is mediated through the use of multiple OB folds [21] (Figure 1A) a common characteristic among single stranded telomere binding proteins such as*Oxytricha nova* TEBP and the vertebrates, plants, and fission yeast Pot1 [22,23]. The first OB fold of Cdc13 (also known as the DNA binding domain (DBD) of Cdc13) was identified by the Wuttke laboratory in 2002 [24]. This domain is centrally located in the protein and has high affinity for single-stranded telomeric DNA [25]. DBD's high affinity and specificity for telomeric DNA facilitates the localization of the full length Cdc13 to the chromosome ends and its stable association with telomeric DNA is most likely essential to enable capping of the chromosomes throughout the cell cycle.

A second Cdc13 OB fold (OB1) identified recently by the Skordalakes laboratory, comprises the N-terminal domain of Cdc13 (Cdc13N). Cdc13N was shown to bind telomeric DNA *in vitro,* supporting earlier *in vivo* evidence from the Zakian laboratory [4]. However, unlike the DBD domain, Cdc13N binds only long segments of single stranded telomeric DNA (≥35 nucleotides) with a K_D_ of around 600 nM [26]. Unexpectedly, Cdc13N was also shown to be involved in Cdc13 dimerization, a characteristic that is necessary for its ability to bind telomeric DNA (Figure 1B). Surprisingly, recent structural data suggests that the pol1 (the catalytic subunit of polα-primase) binding site of Cdc13 overlaps with the DNA binding site of Cdc13N, suggesting that these interactions are mutually-exclusive [27]. It is worth noting that the affinity of pol α for this site is approximately 4 fold lower than that of the telomeric DNA (K_D_ ~2.2 μM as compared to 600 nM for single stranded DNA). Furthermore, the localization of Cdc13 to the telomeres by the DBD domain would position Cdc13N in close proximity to the telomeric overhang, allowing for preferential binding of the telomeric DNA over pol α when long single stranded overhangs are available (i.e., late S phase/G2).

### Telomerase Regulation

Cdc13 acts both as a positive and as a negative regulator of telomerase [28]. It prevents telomerase access to the telomeres through its ability to bind and sequester the telomeric overhang. On the other hand, Cdc13 is essential for recruiting telomerase to the telomeres via its interaction with the telomerase subunit Est1 [29] (Figure 1C). An intriguing question, central to telomere biology, remains: How does telomerase gain access to the telomeric overhang when Cdc13, required for its recruitment to the telomeres, binds and sequesters the very same substrate telomerase needs access to for telomere elongation? The answer to this problem is further compounded by the fact that Est1 competes for the same Cdc13 binding site as Stn1 [25]. These questions have been partially addressed by work carried out by a number of laboratories suggesting that the N-terminal portion of Cdc13 plays a key role in this process. For example, Cdck1 dependent phosphoryla-tion of conserved residues that comprise the recruitment domain (RD) of Cdc13 (Figure 1A) plays a crucial role in the regulation of the telomerase/Stn1 binding to the Cdc13 recruitment domain [30]. However, recent seemingly-conflicting evidence suggests that Stn1 binds to the C-terminal domain of Cdc13, which is located several hundred amino acids away from the recruitment domain [31]. One possibility is that Stn1 binds to both the RD and the C-terminal domains of Cdc13 an arrangement that would require these domains to be in proximity to each other. Dissociation of Stn1 from the RD domain via Cdk1-dependent phosphorylation allows for telomerase binding to this site while Stn1 remains attached to the C-terminal domain of Cdc13. Continuous Stn1 association with the C-terminal domain of Cdc13 would allow for its re-association with the RD when telomeres have been lengthened sufficiently for telomere capping.

### Telomere Length Regulation

Mounting evidence suggests that the N-terminal portion of Cdc13 plays a critical role in telomere length regulation. First, yeast telomerase functions as a dimer [32] and therefore a Cdc13 dimer may facilitate the recruitment of telomerase to the telomeres for elongation. Second, the ability of Cdc13N to weakly bind single stranded telomeric DNA suggests that it can quickly and easily dissociate from the telomeric overhang, thus allowing efficient loading of telomerase to the telomeric overhang for telomere replication. This notion is further supported by the fact that a series of functional assays involving single alanine mutants that disrupt telomere binding by Cdc13N showed a marked increase in telomere length when compared to the wild type protein [26]. Moreover, telomerase recruitment and loading to the telomeric overhang requires that Cdc13 remains attached to the telomeres. This event is most likely facilitated by additional OB folds located downstream of the telomerase recruitment domain of Cdc13, such as the DBD, which has high affinity (3 pM) for telomeric DNA, and therefore can anchor Cdc13 stably to the telomeric overhang (Figure 1D). According to this model, when telomere replication is due to begin, Cdc13N, which is sequestering the telomeric overhang, is induced via its interaction with Hsp82 [31] to release the DNA, thus making it accessible to telomerase for extension. Simultaneously, the Cdc13N DNA binding site becomes accessible for pol α binding thus allowing for C- and G-strand replication (Figure 1D).

### Conclusion

Collectively, structural, biochemical and functional assays suggest that the Cdc13N plays a crucial role in telomere length regulation. Its weak affinity for single-stranded DNA allows Cdc13N to easily dissociate fromthe telomeres (through a mechanism likely dictated by Hsp82) to allow telomerase access to the telomeric overhang for G-strand elongation. At the same time, the overlapping Cdc13N DNA binding site becomes accessible to pol α-primase for binding and recruitment to the telomeres thus facilitating also the replication of the telomeric C-strand. Conversely, the re-association of Cdc13N with the telomere 3'-end terminates both telomerase access to the telomeric overhang and pol α dependent C-strand replication. Further insights into the precise and complex mechanism of action of Cdc13 at the chromosomes ends begs for the structure of the full length Cdc13 alone and in complex with the various protein and nucleic acid substrates that directly associate with this fascinating protein.
